# Bis(1,1,2,2-tetramethyldiphosphane-1,2-dithione-κ^2^ *S*,*S*′)gold(I) trifluoro­methane­sulfonate

**DOI:** 10.1107/S1600536810029326

**Published:** 2010-07-31

**Authors:** Christoph E. Strasser, Stephanie Cronje, Helgard G. Raubenheimer

**Affiliations:** a Department of Chemistry and Polymer Science, University of Stellenbosch, Private Bag X1, Matieland, 7602, South Africa

## Abstract

In the title compound, [Au(C_4_H_12_P_2_S_2_)_2_](CF_3_SO_3_), the gold(I) atom is tightly bonded to two S atoms belonging to different ligand mol­ecules and forms two weaker contacts to the remaining S atoms. The coordination geometry around gold is inter­mediate between linear-dicoordinate and tetra­hedral with an S—Au—S angle of 161.49 (3)°.

## Related literature

For related structure, see: Gimeno *et al.* (2000 ▶); LeBlanc *et al.* (1997 ▶). For complexes of group 11 metals with bidentate diphosphine disulfides, see: Liu *et al.* (2003 ▶).
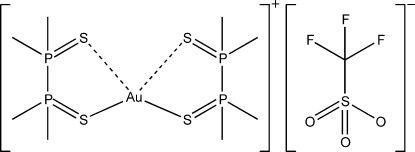


## Experimental

### 

#### Crystal data

[Au(C_4_H_12_P_2_S_2_)_2_](CF_3_O_3_S)
*M* *_r_* = 718.43
Monoclinic, 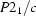

*a* = 13.0115 (10) Å
*b* = 12.6797 (10) Å
*c* = 14.2892 (11) Å
β = 90.800 (1)°
*V* = 2357.2 (3) Å^3^
*Z* = 4
Mo *K*α radiation
μ = 6.99 mm^−1^
*T* = 100 K
0.22 × 0.17 × 0.10 mm

#### Data collection

Bruker APEX CCD area detector diffractometer
Absorption correction: multi-scan (*SADABS*; Bruker, 2002 ▶) *T*_min_ = 0.331, *T*_max_ = 0.542
13530 measured reflections
4790 independent reflections
4513 reflections with *I* > 2σ(*I*)
*R*_int_ = 0.025

#### Refinement

*R*[*F* ^2^ > 2σ(*F* ^2^)] = 0.021
*wR*(*F* ^2^) = 0.052
*S* = 1.05
4790 reflections
234 parameters
H-atom parameters constrained
Δρ_max_ = 1.10 e Å^−3^
Δρ_min_ = −0.63 e Å^−3^

### 

Data collection: *SMART* (Bruker, 2002 ▶); cell refinement: *SAINT* (Bruker, 2003 ▶); data reduction: *SAINT*; program(s) used to solve structure: *SHELXS97* (Sheldrick, 2008 ▶); program(s) used to refine structure: *SHELXL97* (Sheldrick, 2008 ▶); molecular graphics: *X-SEED* (Barbour, 2001 ▶; Atwood & Barbour, 2003 ▶); software used to prepare material for publication: *X-SEED*.

## Supplementary Material

Crystal structure: contains datablocks I, Global. DOI: 10.1107/S1600536810029326/dn2589sup1.cif

Structure factors: contains datablocks I. DOI: 10.1107/S1600536810029326/dn2589Isup2.hkl

Additional supplementary materials:  crystallographic information; 3D view; checkCIF report

## Figures and Tables

**Table 1:** Selected bond lengths (Å)

Au1—S1	2.3099 (7)
Au1—S2	3.3939 (8)
Au1—S3	2.3044 (7)
Au1—S4	3.2472 (8)

**Fig. 1.**
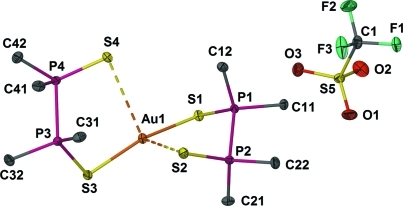
Asymmetric unit of the title compound, ellipsoids are drawn at the 50% probability level.

**Fig. 2.**
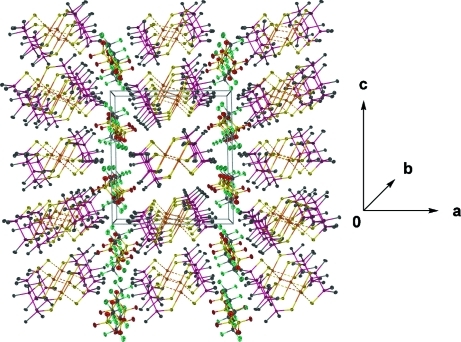
Perspective view of the packing of the title compound along the crystallographic b axis. Ellipsoids are drawn at the 50% probability level, element colours correspond to those used in Figure 1.

## supplementary crystallographic information

### Comment

Complexes of the group 11 metals with bidentate diphosphine disulfides (^2^L) have so far only encompassed the lighter elements Cu and Ag (Liu *et al.*, 2003) which readily yield [M(^2^L)_2_]^+^ cations. Gold complexes have not been investigated, despite the interesting results the combination of these bidentate ligands with the propensity for Au^I^ to form linear-dicoordinate complexes could produce.

The title compound shown in Figure 1 exhibits a structure that is intermediate between classic linear-dicoordinate and tetrahedral coordination. Au^I^ thus forms a complex of the same stoichiometry as its lighter group elements, but with a significantly different geometry. It can be envisaged as a tetrahedron formed by the sulfur donor atoms in which the gold is displaced from the centre towards an edge. The metal is coordinated by two sulfur atoms of different ligands yielding short Au—S bonds [2.3099 (7) and 2.3044 (7) Å] and is further associated with the other sulfur atoms through Au···S contacts [3.3939 (8) and 3.2472 (8) Å]. The attractive nature of these contacts is demonstrated by the S—Au—S angle of 161.49 (3)° which significantly deviates from linearity.

In the molecular structure of the complex [Ag(C_4_H_12_P_2_S_2_)_2_]BF_4_ (Liu *et al.*, 2003) the four Ag—S bonds are more uniform in length with distances between 2.534 (2) and 2.676 (2) Å. The replacement of silver by gold thus causes two of the four bonds to become significantly stronger while the other bonds are reduced to non-classical contacts. Au—S bonds in other phosphine sulfide complexes are significantly shorter than in the present structure, 2.277 (2) Å in [Au(SPPh_3_)_2_]PF_2_O_2_ (LeBlanc *et al.*, 1997) and 2.281 (5) and 2.299 (5) Å in [1,1'-bis(diphenylthiophosphoryl)ferrocene]gold(I) tetrachloroaurate(III) (Gimeno *et al.*, 2000), which can be attributed to the absence of additional stabilizing contacts in these structures.

The [Au(C_4_H_12_P_2_S_2_)_2_]^+^ cations in the crystal structure of the title compound are isolated and no intermolecular Au···Au or Au···S interactions are observed. The packing shown in Figure 2 is characterized by alternating layers of cations and anions parallel to the *bc* plane. The trifluoromethanesulfonate anion shows no disorder and exhibits low thermal movement.

### Experimental

A Schlenk vessel was charged with tetramethyldiphosphine disulfide (129 mg, 0.69 mmol), [AuCl(tht)] (tht = tetrahydrothiophene) (150 mg, 0.47 mmol) and sodium trifluoromethanesulfonate (86 mg, 0.50 mmol). Acetonitrile (20 ml) was added and the suspension stirred for 1 h and evaporated to dryness *in vacuo*. The residue was suspended in acetonitrile (20 ml), filtered, concentrated to *ca* 7 ml and layered with diethyl ether. Crystals grown at 258 K were washed with toluene to remove co-precipitated amorphous solids.

### Refinement

All H atoms were positioned geometrically (C—H = 0.98 Å) and constrained to ride on their parent atoms; *U*_iso_(H) values were set at 1.5 times *U*_eq_(C).

The largest residual electron density peak of 1.10 e Å^-3^ is located 0.83 Å from Au1.

### Crystal data

**Table:**

[Au(C_4_H_12_P_2_S_2_)_2_](CF_3_O_3_S)	*F*(000) = 1392
*M**_r_* = 718.43	*D*_x_ = 2.024 Mg m^−^^3^
Monoclinic, *P*2_1_/*c*	Melting point: 458 K
Hall symbol: -P 2ybc	Mo *K*α radiation, λ = 0.71073 Å
*a* = 13.0115 (10) Å	Cell parameters from 7735 reflections
*b* = 12.6797 (10) Å	θ = 2.7–26.4°
*c* = 14.2892 (11) Å	µ = 6.99 mm^−^^1^
β = 90.800 (1)°	*T* = 100 K
*V* = 2357.2 (3) Å^3^	Plate, colourless
*Z* = 4	0.22 × 0.17 × 0.10 mm

### Data collection

**Table:**

Bruker APEX CCD area detector diffractometer	4790 independent reflections
Radiation source: fine-focus sealed tube	4513 reflections with *I* > 2σ(*I*)
graphite	*R*_int_ = 0.025
ω–scans	θ_max_ = 26.4°, θ_min_ = 2.2°
Absorption correction: multi-scan (*SADABS*; Bruker, 2002)	*h* = −15→16
*T*_min_ = 0.331, *T*_max_ = 0.542	*k* = −15→15
13530 measured reflections	*l* = −16→17

### Refinement

**Table:**

Refinement on *F*^2^	Primary atom site location: structure-invariant direct methods
Least-squares matrix: full	Secondary atom site location: difference Fourier map
*R*[*F*^2^ > 2σ(*F*^2^)] = 0.021	Hydrogen site location: inferred from neighbouring sites
*wR*(*F*^2^) = 0.052	H-atom parameters constrained
*S* = 1.05	*w* = 1/[σ^2^(*F*_o_^2^) + (0.0282*P*)^2^] where *P* = (*F*_o_^2^ + 2*F*_c_^2^)/3
4790 reflections	(Δ/σ)_max_ = 0.002
234 parameters	Δρ_max_ = 1.10 e Å^−^^3^
0 restraints	Δρ_min_ = −0.63 e Å^−^^3^

### Special details

**Table:**

Geometry. All e.s.d.'s (except the e.s.d. in the dihedral angle between two l.s. planes) are estimated using the full covariance matrix. The cell e.s.d.'s are taken into account individually in the estimation of e.s.d.'s in distances, angles and torsion angles; correlations between e.s.d.'s in cell parameters are only used when they are defined by crystal symmetry. An approximate (isotropic) treatment of cell e.s.d.'s is used for estimating e.s.d.'s involving l.s. planes.
Refinement. Refinement of *F*^2^ against ALL reflections. The weighted *R*-factor *wR* and goodness of fit *S* are based on *F*^2^, conventional *R*-factors *R* are based on *F*, with *F* set to zero for negative *F*^2^. The threshold expression of *F*^2^ > 2σ(*F*^2^) is used only for calculating *R*-factors(gt) *etc*. and is not relevant to the choice of reflections for refinement. *R*-factors based on *F*^2^ are statistically about twice as large as those based on *F*, and *R*- factors based on ALL data will be even larger.

### Fractional atomic coordinates and isotropic or equivalent isotropic displacement parameters (Å^2^)

**Table:**

	*x*	*y*	*z*	*U*_iso_*/*U*_eq_	
Au1	0.440979 (8)	0.844393 (8)	0.524100 (8)	0.01389 (5)	
S1	0.52186 (5)	0.92223 (5)	0.65138 (5)	0.01472 (15)	
S2	0.63626 (6)	0.67687 (6)	0.47459 (5)	0.01673 (15)	
S3	0.35744 (6)	0.82264 (6)	0.38215 (5)	0.01501 (15)	
S4	0.32851 (6)	0.64875 (6)	0.62763 (6)	0.01872 (17)	
P1	0.64439 (6)	0.82795 (6)	0.67067 (5)	0.01163 (15)	
P2	0.71405 (5)	0.79124 (6)	0.53285 (5)	0.01190 (15)	
P3	0.29943 (6)	0.67616 (6)	0.39245 (5)	0.01318 (15)	
P4	0.22373 (6)	0.65658 (5)	0.52912 (6)	0.01294 (16)	
C11	0.7375 (2)	0.8935 (2)	0.7437 (2)	0.0171 (6)	
H11A	0.7055	0.9144	0.8026	0.026*	
H11B	0.7629	0.9564	0.7115	0.026*	
H11C	0.7950	0.8456	0.7571	0.026*	
C12	0.6124 (2)	0.7039 (2)	0.7218 (2)	0.0164 (6)	
H12A	0.6746	0.6609	0.7284	0.025*	
H12B	0.5624	0.6673	0.6814	0.025*	
H12C	0.5826	0.7155	0.7835	0.025*	
C21	0.7118 (2)	0.9145 (2)	0.4715 (2)	0.0186 (6)	
H21A	0.7465	0.9684	0.5095	0.028*	
H21B	0.6404	0.9357	0.4596	0.028*	
H21C	0.7472	0.9067	0.4118	0.028*	
C22	0.8470 (2)	0.7616 (2)	0.5572 (2)	0.0186 (6)	
H22A	0.8827	0.7493	0.4983	0.028*	
H22B	0.8516	0.6983	0.5964	0.028*	
H22C	0.8790	0.8211	0.5903	0.028*	
C31	0.3938 (2)	0.5739 (2)	0.3907 (2)	0.0195 (6)	
H31A	0.3598	0.5051	0.3952	0.029*	
H31B	0.4415	0.5828	0.4438	0.029*	
H31C	0.4319	0.5776	0.3321	0.029*	
C32	0.2097 (3)	0.6538 (2)	0.2981 (2)	0.0213 (7)	
H32A	0.2420	0.6714	0.2386	0.032*	
H32B	0.1490	0.6983	0.3066	0.032*	
H32C	0.1890	0.5795	0.2976	0.032*	
C41	0.1386 (2)	0.7673 (2)	0.5343 (2)	0.0198 (6)	
H41A	0.0943	0.7683	0.4783	0.030*	
H41B	0.1789	0.8326	0.5373	0.030*	
H41C	0.0961	0.7617	0.5901	0.030*	
C42	0.1452 (2)	0.5404 (2)	0.5155 (2)	0.0219 (7)	
H42A	0.1006	0.5331	0.5699	0.033*	
H42B	0.1894	0.4781	0.5108	0.033*	
H42C	0.1028	0.5468	0.4586	0.033*	
F1	1.09451 (13)	0.54274 (14)	0.87757 (13)	0.0238 (4)	
F2	0.93924 (14)	0.50519 (17)	0.91581 (15)	0.0390 (5)	
F3	1.00332 (15)	0.43955 (15)	0.79097 (16)	0.0387 (5)	
C1	0.9995 (2)	0.5259 (2)	0.8438 (2)	0.0202 (6)	
S5	0.95244 (6)	0.63932 (6)	0.77742 (6)	0.01722 (16)	
O1	1.02253 (19)	0.64635 (19)	0.70158 (18)	0.0325 (6)	
O2	0.95933 (18)	0.72418 (18)	0.84446 (16)	0.0319 (6)	
O3	0.84886 (15)	0.60862 (18)	0.75253 (16)	0.0268 (5)	

### Atomic displacement parameters (Å^2^)

**Table:**

	*U*^11^	*U*^22^	*U*^33^	*U*^12^	*U*^13^	*U*^23^
Au1	0.01265 (7)	0.01445 (7)	0.01452 (7)	0.00035 (4)	−0.00187 (5)	−0.00070 (4)
S1	0.0147 (3)	0.0143 (3)	0.0151 (4)	0.0019 (3)	−0.0008 (3)	−0.0025 (3)
S2	0.0184 (4)	0.0175 (3)	0.0142 (4)	−0.0030 (3)	−0.0009 (3)	−0.0036 (3)
S3	0.0163 (4)	0.0145 (3)	0.0142 (4)	−0.0032 (3)	−0.0027 (3)	0.0017 (3)
S4	0.0183 (4)	0.0235 (4)	0.0142 (4)	−0.0013 (3)	−0.0033 (3)	0.0026 (3)
P1	0.0115 (3)	0.0131 (3)	0.0103 (4)	−0.0004 (3)	−0.0005 (3)	−0.0008 (3)
P2	0.0123 (3)	0.0125 (3)	0.0110 (4)	0.0006 (3)	0.0008 (3)	0.0003 (3)
P3	0.0140 (4)	0.0139 (3)	0.0117 (4)	−0.0026 (3)	−0.0002 (3)	−0.0004 (3)
P4	0.0131 (4)	0.0129 (4)	0.0129 (4)	−0.0014 (3)	0.0009 (3)	0.0009 (3)
C11	0.0150 (14)	0.0216 (15)	0.0146 (15)	−0.0046 (12)	−0.0016 (12)	−0.0025 (12)
C12	0.0189 (15)	0.0147 (14)	0.0155 (15)	0.0012 (12)	−0.0007 (12)	0.0027 (11)
C21	0.0233 (15)	0.0163 (15)	0.0163 (16)	−0.0004 (12)	0.0040 (13)	0.0026 (12)
C22	0.0131 (14)	0.0243 (16)	0.0184 (16)	0.0014 (12)	−0.0024 (12)	−0.0010 (13)
C31	0.0221 (15)	0.0149 (15)	0.0217 (16)	−0.0003 (12)	0.0065 (13)	0.0006 (12)
C32	0.0255 (18)	0.0246 (18)	0.0138 (16)	−0.0068 (12)	−0.0049 (14)	−0.0019 (12)
C41	0.0213 (15)	0.0192 (16)	0.0189 (16)	0.0045 (12)	0.0036 (13)	0.0019 (12)
C42	0.0205 (16)	0.0228 (16)	0.0225 (17)	−0.0074 (13)	0.0000 (13)	0.0009 (13)
F1	0.0159 (9)	0.0270 (10)	0.0282 (10)	0.0025 (7)	−0.0078 (8)	−0.0023 (8)
F2	0.0261 (11)	0.0484 (13)	0.0425 (13)	0.0023 (9)	0.0034 (9)	0.0292 (11)
F3	0.0340 (11)	0.0209 (10)	0.0605 (15)	0.0079 (8)	−0.0227 (10)	−0.0133 (10)
C1	0.0150 (14)	0.0191 (15)	0.0263 (17)	0.0015 (12)	−0.0048 (13)	0.0015 (13)
S5	0.0147 (4)	0.0196 (4)	0.0174 (4)	0.0021 (3)	0.0008 (3)	0.0052 (3)
O1	0.0239 (13)	0.0513 (17)	0.0226 (14)	0.0040 (10)	0.0080 (11)	0.0127 (11)
O2	0.0419 (15)	0.0237 (13)	0.0302 (14)	0.0075 (11)	−0.0031 (11)	−0.0039 (10)
O3	0.0164 (11)	0.0299 (13)	0.0339 (14)	0.0012 (9)	−0.0052 (10)	0.0097 (11)

### Geometric parameters (Å, °)

**Table:**

Au1—S1	2.3099 (7)	C21—H21B	0.9800
Au1—S2	3.3939 (8)	C21—H21C	0.9800
Au1—S3	2.3044 (7)	C22—H22A	0.9800
Au1—S4	3.2472 (8)	C22—H22B	0.9800
S1—P1	2.0086 (10)	C22—H22C	0.9800
S2—P2	1.9486 (10)	C31—H31A	0.9800
S3—P3	2.011 (1)	C31—H31B	0.9800
S4—P4	1.9483 (12)	C31—H31C	0.9800
P1—P2	2.2285 (10)	C32—H32A	0.9800
P1—C11	1.793 (3)	C32—H32B	0.9800
P1—C12	1.786 (3)	C32—H32C	0.9800
P2—C21	1.792 (3)	C41—H41A	0.9800
P2—C22	1.799 (3)	C41—H41B	0.9800
P3—P4	2.2135 (11)	C41—H41C	0.9800
P3—C31	1.787 (3)	C42—H42A	0.9800
P3—C32	1.793 (3)	C42—H42B	0.9800
P4—C41	1.791 (3)	C42—H42C	0.9800
P4—C42	1.802 (3)	F1—C1	1.338 (3)
C11—H11A	0.9800	F2—C1	1.329 (4)
C11—H11B	0.9800	F3—C1	1.331 (4)
C11—H11C	0.9800	C1—S5	1.823 (3)
C12—H12A	0.9800	S5—O1	1.429 (2)
C12—H12B	0.9800	S5—O2	1.443 (2)
C12—H12C	0.9800	S5—O3	1.443 (2)
C21—H21A	0.9800		
			
S1—Au1—S2	95.59 (2)	P2—C21—H21B	109.5
S1—Au1—S3	161.49 (3)	H21A—C21—H21B	109.5
S1—Au1—S4	99.84 (2)	P2—C21—H21C	109.5
S2—Au1—S3	94.99 (2)	H21A—C21—H21C	109.5
S2—Au1—S4	87.73 (2)	H21B—C21—H21C	109.5
S3—Au1—S4	95.71 (2)	P2—C22—H22A	109.5
P1—S1—Au1	101.88 (4)	P2—C22—H22B	109.5
P2—S2—Au1	80.28 (3)	H22A—C22—H22B	109.5
P3—S3—Au1	102.65 (4)	P2—C22—H22C	109.5
P4—S4—Au1	86.97 (3)	H22A—C22—H22C	109.5
S1—P1—P2	109.57 (4)	H22B—C22—H22C	109.5
S2—P2—P1	108.53 (4)	P3—C31—H31A	109.5
C11—P1—S1	109.42 (10)	P3—C31—H31B	109.5
C11—P1—P2	109.37 (10)	H31A—C31—H31B	109.5
C12—P1—S1	113.03 (10)	P3—C31—H31C	109.5
C12—P1—C11	109.26 (14)	H31A—C31—H31C	109.5
C12—P1—P2	106.1 (1)	H31B—C31—H31C	109.5
C21—P2—C22	106.63 (14)	P3—C32—H32A	109.5
C21—P2—S2	115.78 (11)	P3—C32—H32B	109.5
C21—P2—P1	104.25 (10)	H32A—C32—H32B	109.5
C22—P2—S2	114.80 (11)	P3—C32—H32C	109.5
C22—P2—P1	105.92 (10)	H32A—C32—H32C	109.5
S3—P3—P4	109.89 (4)	H32B—C32—H32C	109.5
S4—P4—P3	109.13 (5)	P4—C41—H41A	109.5
C31—P3—S3	114.25 (10)	P4—C41—H41B	109.5
C31—P3—C32	108.39 (15)	H41A—C41—H41B	109.5
C31—P3—P4	104.2 (1)	P4—C41—H41C	109.5
C32—P3—S3	109.38 (10)	H41A—C41—H41C	109.5
C32—P3—P4	110.63 (11)	H41B—C41—H41C	109.5
C41—P4—S4	115.93 (11)	P4—C42—H42A	109.5
C42—P4—S4	115.23 (11)	P4—C42—H42B	109.5
C41—P4—P3	103.38 (10)	H42A—C42—H42B	109.5
C41—P4—C42	107.16 (16)	P4—C42—H42C	109.5
C42—P4—P3	104.80 (11)	H42A—C42—H42C	109.5
P1—C11—H11A	109.5	H42B—C42—H42C	109.5
P1—C11—H11B	109.5	F1—C1—F2	107.7 (3)
H11A—C11—H11B	109.5	F1—C1—F3	107.1 (2)
P1—C11—H11C	109.5	F2—C1—F3	107.6 (3)
H11A—C11—H11C	109.5	F1—C1—S5	111.4 (2)
H11B—C11—H11C	109.5	F2—C1—S5	111.2 (2)
P1—C12—H12A	109.5	F3—C1—S5	111.6 (2)
P1—C12—H12B	109.5	O1—S5—O2	115.00 (16)
H12A—C12—H12B	109.5	O1—S5—O3	115.71 (15)
P1—C12—H12C	109.5	O2—S5—O3	114.47 (15)
H12A—C12—H12C	109.5	O1—S5—C1	103.30 (14)
H12B—C12—H12C	109.5	O2—S5—C1	103.04 (15)
P2—C21—H21A	109.5	O3—S5—C1	102.80 (14)
			
S3—Au1—S1—P1	−134.62 (7)	S1—P1—P2—S2	−81.71 (5)
S4—Au1—S1—P1	78.61 (4)	Au1—S3—P3—C31	−69.84 (12)
S2—Au1—S1—P1	−10.05 (4)	Au1—S3—P3—C32	168.48 (12)
S3—Au1—S2—P2	136.79 (3)	Au1—S3—P3—P4	46.85 (5)
S1—Au1—S2—P2	−28.00 (4)	Au1—S4—P4—C41	−72.22 (12)
S4—Au1—S2—P2	−127.67 (3)	Au1—S4—P4—C42	161.49 (12)
S1—Au1—S3—P3	−163.26 (7)	Au1—S4—P4—P3	43.95 (4)
S4—Au1—S3—P3	−16.12 (4)	C31—P3—P4—C41	174.52 (15)
S2—Au1—S3—P3	72.08 (4)	C32—P3—P4—C41	−69.19 (15)
S3—Au1—S4—P4	−18.69 (4)	S3—P3—P4—C41	51.70 (12)
S1—Au1—S4—P4	151.24 (3)	C31—P3—P4—C42	−73.35 (15)
S2—Au1—S4—P4	−113.48 (3)	C32—P3—P4—C42	42.94 (15)
Au1—S1—P1—C12	−74.47 (11)	S3—P3—P4—C42	163.82 (11)
Au1—S1—P1—C11	163.53 (10)	C31—P3—P4—S4	50.59 (11)
Au1—S1—P1—P2	43.62 (5)	C32—P3—P4—S4	166.88 (11)
Au1—S2—P2—C21	−64.82 (11)	S3—P3—P4—S4	−72.24 (6)
Au1—S2—P2—C22	170.20 (11)	F2—C1—S5—O1	−177.2 (2)
Au1—S2—P2—P1	51.93 (4)	F3—C1—S5—O1	−57.0 (2)
C12—P1—P2—C21	164.56 (14)	F1—C1—S5—O1	62.7 (2)
C11—P1—P2—C21	−77.71 (15)	F2—C1—S5—O3	−56.5 (3)
S1—P1—P2—C21	42.23 (11)	F3—C1—S5—O3	63.7 (2)
C12—P1—P2—C22	−83.14 (15)	F1—C1—S5—O3	−176.6 (2)
C11—P1—P2—C22	34.60 (15)	F2—C1—S5—O2	62.7 (3)
S1—P1—P2—C22	154.53 (11)	F3—C1—S5—O2	−177.1 (2)
C12—P1—P2—S2	40.61 (11)	F1—C1—S5—O2	−57.4 (2)
C11—P1—P2—S2	158.35 (11)		
